# Implementation of Artificial Neural Network to Predict Diabetes with High-Quality Health System

**DOI:** 10.1155/2022/1174173

**Published:** 2022-05-30

**Authors:** Prakash E. P., Srihari K., S. Karthik, Kamal M. V., Dileep P., Bharath Reddy S., Mukunthan M. A., Somasundaram K., Jaikumar R., Gayathri N., Kibebe Sahile

**Affiliations:** ^1^Department of Computer Science & Engineering, SNS College of Engineering, Coimbatore 641107, Tamilnadu, India; ^2^Department of Computer Science and Engineering, Malla Reddy College of Engineering and Technology, Kompally, Hyderabad, India; ^3^AIML College, Vardhaman College of Engineering, Shamshabad, Hyderabad, India; ^4^Department of Computer Science and Engineering, VELTECH Science and Technology University, Avadi, Chennai 71, India; ^5^Institute of Information Technology, Saveetha School of Engineering, SIMATS, Thandalam, Chennai 602 105, Tamilnadu, India; ^6^Department of ECE, KGiSL Institute of Technology, Coimbatore, India; ^7^Veltech High Tech Dr. Rangarajan Dr. Sakunthala Engineering College, Chennai, India; ^8^Department of Chemical Engineering, College of Biological and Chemical Engineering, Addis Ababa Science and Technology University, Addis Ababa, Ethiopia

## Abstract

Patients with diabetes who are closely monitored have a higher overall quality of life than those who are not. Costs associated with healthcare can be decreased by utilising the Internet of Things (IoT), thanks to technological advancements. To satisfy the expectations of e-health applications, it is required for the development of the intelligent systems as well as increases the number of applications that are connected to the network. As a result, in order to achieve these goals, the cellular network should be capable of supporting intelligent healthcare applications that require high energy efficiency. In this paper, we model a neural network-based ensemble voting classifier to predict accurately the diabetes in the patients via online monitoring. The study consists of Internet of Things (IoT) devices to monitor the instances of the patients. While monitoring, the data are transferred from IoT devices to smartphones and then to the cloud, where the process of classification takes place. The simulation is conducted on the collected samples using the python tool. The results of the simulation show that the proposed method achieves a higher accuracy rate, higher precision, recall, and f-measure than existing state-of-art ensemble models.

## 1. Introduction

In the rapidly evolving field of healthcare, a diverse range of research possibilities are available. The Internet of Things (IoT) is driving this transformation in the way things are done, and it will continue to do so (IoT). Patients with chronic illnesses are the primary benefactors of modern technology, which has gained in popularity in recent years due to its ability to treat them more effectively. As a result, Internet of Things technology offers diabetes patients new options.

Chronic diseases, on the other hand, are long-lasting and necessitate long-term treatment. The majority of patients with long-term medical illnesses, such as cancer or heart disease, are admitted to the hospital and remain there for significant periods of time. Chronic diseases such as heart disease, cancer, and diabetes are among the most common. Diabetes is today a very severe problem since it is the leading cause of death in the United States, killing thousands of individuals each year. In order to operate normally on a daily basis, the diabetic patient must be properly treated on an ongoing basis.

Diabetes is considered as a long-term condition that tends to occur if it fails to produce insulin [1]. Blood sugar levels (high or low) can have a negative impact on a variety of organs, including the eyes, neurons, and blood vessels, among others. Patients with diabetes must have their health regularly monitored in order to prevent their health from deteriorating.

The rise in the number of diabetic patients has necessitated an increase in the use of patient monitoring devices during the last few years. The purpose of diabetic patient monitoring systems is to continuously monitor blood glucose levels in diabetic patients. The glucose levels can be monitored at all times by patients, family members, and doctors, who can respond quickly if anything appears to be amiss.

The life of patients with diabetes is enhanced by reducing the length of their hospital stays, which can be accomplished through the use of diabetic portable monitoring devices. The use of wireless technology to communicate data between patients and healthcare providers that has a wide range of coverage could be particularly beneficial in these instances, according to the researchers. High-speed transmission as well as network capacity and scalability in the next general cellular network technology are the most important features of 5G technology. Data transfer speeds are currently being evaluated using this technology [2] to see if it has the potential to speed up data transport.

The use of deterministic mathematical models in the treatment of diabetic patients can be beneficial. There has been little attention paid to mathematical models of diabetes mellitus in the scientific literature to date. When it comes to determining the pandemic potential of diabetes mellitus, a technique known as stochastic numerical analysis (SNA) is still relevant [3, 4].

In our technique, data collected by a simple sensor with low cost and power was employed for categorization purposes. The patient information was updated on a daily basis in the cloud. It is possible that doctors will use this information to detect and treat diabetes in patients whose blood glucose levels fluctuate outside of the usual range.

For the purpose of obtaining the result that was obtained, a number of machine learning approaches were applied. The analysis, testing, and comparison of many categorization algorithms were carried out for providing the highest level of accuracy feasible for the classification.

In this paper, we developed a smart continuous monitoring architecture for monitoring the diabetic patients using the Internet of Things (IoT), which was based on machine learning categorization and the IoT. Patient blood sugar, temperature, and exercise levels were all monitored by portable sensors that were included in our design. Data collection and analysis were carried out using a number of different categorization strategies. The proposed technology also provided diabetes patients with the capability of predicting their blood sugar levels in the future, which was advantageous to them.

## 2. Background

In this section, we conducted a review of previously published research on 5G-based solutions for diabetic patient blood glucose level monitoring, as well as possible future research directions [4]. The next part also contains some previously published works that use classification to forecast blood sugar spikes and falls using big data and predictive analytics, which are included in this section. The classification of e-health monitoring data is crucial to the success of treatment.

Bukhari et al. [5] examine smart healthcare applications that are enabled by 5G and the Internet of Things. The authors [6] describe how healthcare delivery via 5G has its own set of barriers, research trends, and potential future paths, all of which are unique to this field.

Chen et al. [7] propose a health system that takes advantage of 5G technology to continuously evaluate and monitor diabetes patients in order to provide diabetic patients with comprehensive monitoring and analysis services. Because of this, the authors present the data exchange mechanism and approach used for 5G-Smart Diabetes data processing and visualisation. Researchers also used a 5G-Smart Diabetes test bed to gather data. In accordance with the results, patients can be diagnosed and treated using the approach described [8].

The system that takes advantage of the IoT, smart home monitoring is developed by Xiao et al. [8]. For blind users to avoid obstacles, the received signal strength indicator (RSSI) is extracted, which were then used to avoid obstacles. Experiments with this technology revealed that it avoided obstacles with a 94 percent accuracy rate.

According to a study conducted by Chatrati et al. [9], a monitoring system helps in the prediction of type 2 diabetes and high blood pressure. This technology allows patients to monitor their blood pressure and glucose levels in the comfort of their own homes. In the event of an irregularity, the caregiver is notified as soon as possible. High blood pressure and diabetes can also be predicted using supervised machine learning classification algorithms, as can other chronic diseases.

A novel machine learning approach based on the decision tree (DT) method is developed by Najm et al. [10] to estimate the best improvement in congestion control for 5G IoT wireless sensors using decision trees. Our model's primary objective in a 5G context is to determine what the optimal parametric configuration is for that environment.

According to their findings, the glucose levels of diabetic individuals can be predicted using a new technique presented by Ahmed and Serener [11]. The authors conducted their analysis of patient data using the GlucoSim programme. The use of a CGS and a KF (Kalman Filter) in this system helps to limit the amount of noise produced by it. With this method, hypo- and hyperglycemia, which can lead to life-threatening complications, can be avoided.

The classification of the Pima Indian diabetes dataset is the goal of Kannadasan et al. [12]. The authors [13, 14] propose stacking autoencoders as a deep neural network architecture for the classification of diabetes data, and they demonstrate its effectiveness in this study.

A model for personalised heart condition categorization is presented by Yoo et al. [15], which is combined with a rapid and effective preprocessing technique and a deep neural network classifier for analysing the data obtained from the biosensor.

Valenzuela et al. [16] developed a monitoring system using handoff protocols. The two-tier architecture of this system includes a layer of worn sensors for gathering vital signs and a layer of body sensor network coordinator devices. While wearing the sensor on one wrist and walking at a pace of 0.55 m/s, the best results were obtained, with the loss rate being 20% lower than when using a point-to-point coordinator–AP (access point) link between two locations.

As the last stage, we examine a number of pieces of research that make use of machine learning techniques. According to Izonin et al. [17], support vector machines and Wiener polynomials can be employed to solve the classification problem of medical implants. The authors compare and contrast his ideas with techniques that have already been established.

One of the most important objectives of our research was the development of a revolutionary. It was not discussed in any of the studies reviewed here that employing machine learning techniques to categorise data from diabetes patients while using a cellular network infrastructure was a viable option [18, 19]. Our strategy is detailed based on patients with diabetes, and their associated parameters are not the only factors that may be analysed by our system. Therefore, we have concentrated on employing a variety of machine learning approaches to classify this data in order to save time [20, 21].

## 3. Proposed Method

**Figure 1:**
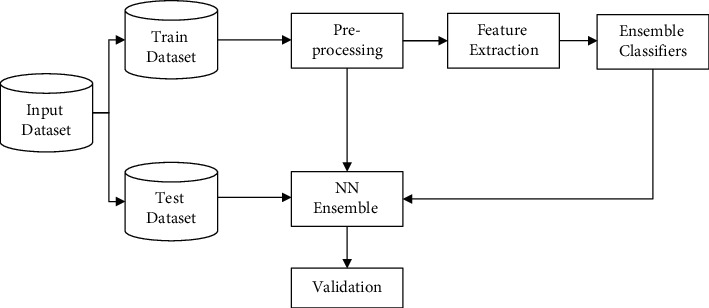
Proposed classification model.

In this section, a classification model is built using ensemble machine learning models that initially involve a series of operations that include preprocessing, feature extraction, and then the training and testing of individual machine learning classifiers as given in Figure 1.

Diabetes patients' blood glucose levels, body temperature, and physical activity were to be tracked using an Internet of Things sensor, with the data being relayed to a base station after the fact. Then, in order to aid users in maintaining their glucose levels and forecasting future health changes, the system intelligently processed the data using artificial intelligence and machine learning technologies.

When it comes to diabetic individuals, it is critical that their blood glucose levels are regularly checked because even minor fluctuations can have serious consequences for their health, resulting in a diabetic coma, blindness, and even death if they occur frequently. As part of their diabetes management regimen, diabetics are well aware that they must take insulin on a daily basis. As a result, a remote system for home blood glucose monitoring and prompt intervention was proposed as a solution to this problem. Patient doctors would be warned if they entered incorrect values into the system. Based on this information, the doctors were able to prescribe specific treatment programmes for the patients.

### 3.1. Ensemble Classification Model

**Figure 2:**
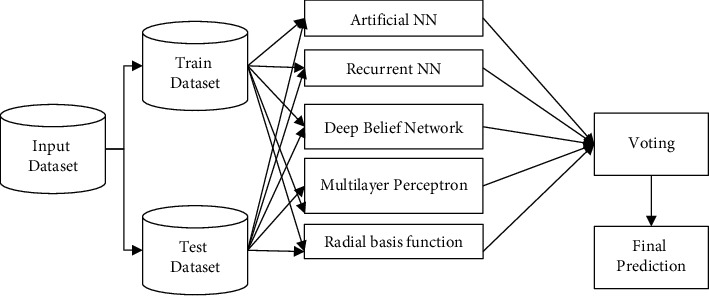
Ensemble classification model.

In this section, we use different neural network models including the artificial neural network model, recurrent neural network model, deep belief network, multilayer perceptron, and radial basis function as the base classifier. The results that are used for predicting the class based on the higher level of probability of the class are considered as its output. The illustration is given in Figure 2.

It is possible to merge the majority of machine learning models into a single predictive model with the help of a majority vote. It is necessary for the voting classifier to use both hard and soft voting methods in order to classify votes. In order to make the final forecast, aggregators employ hard voting to select the class prediction that appears repeatedly among base models in order to make the final prediction. It is recommended that the predict probability technique be included in all base models that are used for soft voting. The voting classifier achieves better overall results than other base models by combining predictions from a large number of models.

According to the results, a recurrent neural network or deep belief network gives the highest probability of the classes; all other networks have lower probabilities. As long as the probability of each voter being correct is greater than 0.5 and the voters are independent, increasing the number of voters increases the probability of a majority vote being correct until the probability of the majority vote being correct reaches a level that is statistically insignificant.

This technology has demonstrated promising results in a number of fields, including object detection, semantic segmentation, and a variety of other applications. The training of deep ensemble models, on the other hand, is a difficult task due to the enormous computing costs involved. Several viewpoints have been presented in order to better understand how deep learning models learn features such as hierarchical notions through multiple layers of representation.

There are a number of advantages to deep learning models, such as vanishing or expanding gradients and degradation difficulties that make it harder to attain this goal using them. Both of these networks might be used to train networks with this level of depth. Deep learning models can benefit substantially from the use of ensemble learning, which has only recently been found and is still in its early stages. Because of this, one of the primary goals of employing deep ensemble models is to develop a model that contains the best characteristics of both ensemble and deep models.

When combining the output of the base predictors, the method used to do so may differ depending on the type of classification problem being solved. However, in regression circumstances, the majority voting approach is most typically used to combine the output. The outputs of the various models are aggregated using a combination of methods, including majority voting, least squares estimation weighting, and a two-layer hierarchical methodology. When using the double-layer hierarchical technique, a second neural network is used to aggregate the outputs of several neural networks more quickly and effectively.

Following the combination of several classifiers into one, we were successful in creating an ensemble classifier with soft voting. This classifier makes decisions on the basis of the probability values associated with each individual decision it encounters. In the soft voting ensemble approach, predictions are weighted according to the importance of the classifier, and the weighted probabilities are added together to provide the total of weighted probabilities.

Ultimately, the target label with the greatest total of weighted probabilities is selected as a result of this procedure. Custom weights can also be used to create the weighted average, which can be used to give more weight to a specific learning model. Soft voting outperforms hard voting because it makes use of the averaging of probabilities to produce a more accurate outcome and greater performance.

In order to compensate for the limitations of individual base classifiers, the soft voting ensemble classifier aggregates a large number of prediction models, outperforming the overall set of results. One of the key goals of ensemble techniques is to reduce the amount of bias and volatility.

## 4. Results and Discussion

In this section, the entire simulation is conducted using python tool on a high-end computing system that involves the computation of the Pima Indians diabetes dataset for training and testing the classifier.

**Figure 3:**
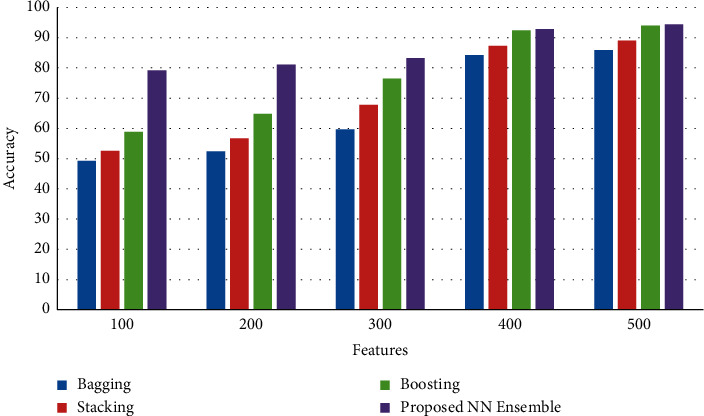
Accuracy.

Figure 3 shows the results of accuracy between the proposed neural network ensemble voting method with existing bagging, stacking, and boosting ensemble models. The results of the simulation show that the proposed neural network ensemble voting method achieves an improved rate of accuracy than existing bagging, stacking, and boosting ensemble models.

**Figure 4:**
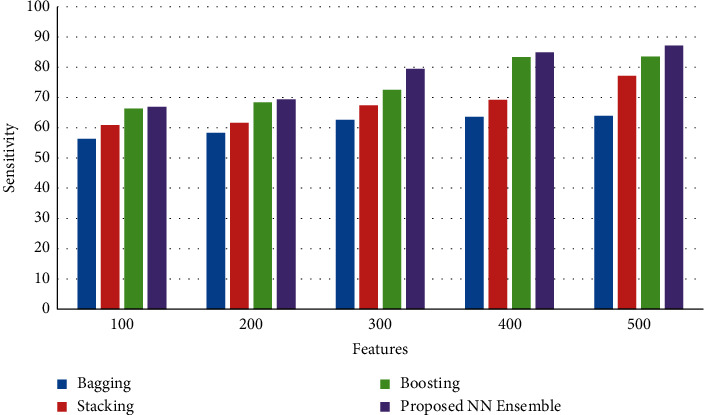
Sensitivity.

Figure 4 shows the results of sensitivity between the proposed neural network ensemble voting method and existing bagging, stacking, and boosting ensemble models. The results of the simulation show that the proposed neural network ensemble voting method achieves an improved rate of sensitivity than existing bagging, stacking, and boosting ensemble models.

**Figure 5:**
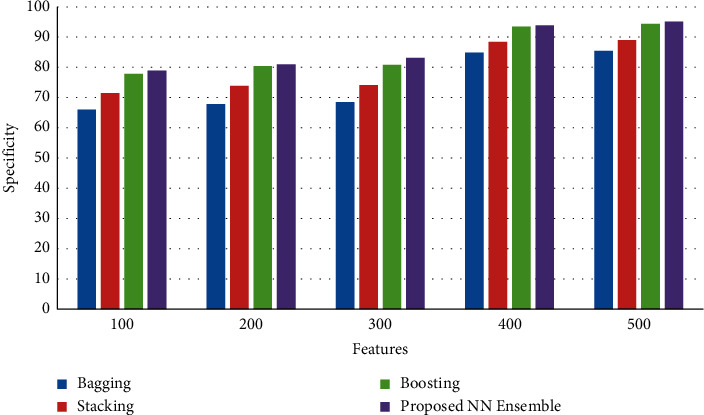
Specificity.

Figure 5 shows the results of specificity between the proposed neural network ensemble voting method and existing bagging, stacking, and boosting ensemble models. The results of the simulation show that the proposed neural network ensemble voting method achieves an improved rate of specificity than existing bagging, stacking, and boosting ensemble models.

**Figure 6:**
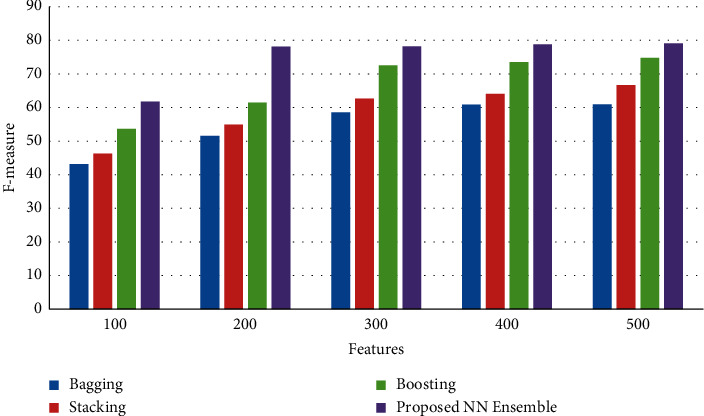
F-measure.

Figure 6 shows the results of F-measure between the proposed neural network ensemble voting method and existing bagging, stacking, and boosting ensemble models. The results of the simulation show that the proposed neural network ensemble voting method achieves an improved rate of F-measure than existing bagging, stacking, and boosting ensemble models.

## 5. Conclusions

Predictive analytics can be used in the healthcare industry to gain insights from medical data and make better decisions, among other things. The development of a diabetic patient monitoring system for this inquiry was made possible by the employment of machine learning algorithms. We propose an approach for accurately predicting the presence of diabetes in patients, which makes use of online monitoring and a neural network ensemble voting classifier. The study makes use of IoT devices to track the actions and outcomes of the patients. Classification takes place on the cloud after data from IoT devices and smartphones have been submitted to the system. Simulation results show that the proposed method achieves a higher degree of accuracy with 99% than other existing methods.

## Data Availability

The datasets used and/or analysed during the current study are available from the corresponding author on reasonable request.
